# “Breaking barriers: empowering African youth through implementation of a therapeutic patient education program for juvenile idiopathic arthritis”

**DOI:** 10.3389/fped.2024.1479857

**Published:** 2024-12-03

**Authors:** Wafa Hamdi, Angela Migowa, Hanene Lassoued Ferjani, Alia Fazaa, Ayodele Faleye, Saoussen Miladi, Kaouther Maatallah, Kawther Ben Abdelghani

**Affiliations:** ^1^ Department of Rheumatology, Kassab Institute, Faculty of Medicine of Tunis, Tunis El Manar University, Tunis, Tunisia; ^2^ Department of Paediatrics and Child Health, Aga Khan University Medical College East Africa, Nairobi, Kenya; ^3^ Department of Rheumatology, Mongi Slim Hospital, Faculty of Medicine of Tunis, Tunis El Manar University, Tunis, Tunisia; ^4^ Paediatric Rheumatology Unit, Department of Paediatrics, Lagos State University Teaching Hospital, Ikeja, Lagos, Nigeria

**Keywords:** juvenile idiopathic arthritis, patient centered care, therapeutic patient education, disease self-management, train the trainers

## Abstract

**Introduction:**

Patient education is crucial in managing chronic diseases like Juvenile Idiopathic Arthritis (JIA). Traditional methods such as videos and brochures often fail to maintain long-term knowledge retention. Therapeutic Patient Education (TPE) offers a more effective, patient-centered approach.

**Objective:**

PAFLAR aimed to implement a TPE program with a “train the trainers” masterclass to ensure effective delivery and improve outcomes for children with JIA.

**Methods:**

PAFLAR's TPE program was designed through a focus group, involving a literature review and strategic planning for the implementation step. The program includes specialized training for healthcare professionals enhancing their abilities to deliver TPE workshops. Participants were selected based on volunteerism and commitment to the project. Evaluations were conducted through feedback assessment of both patients and participants.

**Results:**

PAFLAR launched a TPE program in 2023, aimed at training healthcare providers to implement TPE effectively. This initiative included both in-person and virtual masterclasses, resulting in five workshops conducted in Kenya, Tunisia, and Nigeria. These workshops covered various aspects of JIA management, such as patient and family education, self-esteem, physical activity, treatment adherence, and pain management. Early results showed significant improvements in patients’ and parents’ understanding and management of JIA, with positive feedback indicating a need for further sessions.

**Conclusion:**

PAFLAR has taken the initial step in implementing a TPE program for JIA across three countries, representing different regions of Africa. The TPE program offers a promising alternative to traditional patient education methods, significantly improving patient care, empowering healthcare providers, and advancing healthcare systems.

## Introduction

The prevalence of Juvenile Idiopathic Arthritis (JIA) in Africa seems to fall on the lower end of global estimates. However, there remains a significant unmet need in the region for accurate epidemiological data, increased disease awareness, and improved quality of care in the management of JIA (1).

Patient education is essential for managing chronic diseases like JIA (2, 3). Various tools, such as brochures and videos, are used to achieve educational goals. Research shows that while videos can significantly enhance JIA knowledge immediately after viewing, especially for those with lower education levels, knowledge retention decreases after four weeks, highlighting the limitations of passive methods (4, 5).

Therapeutic Patient Education (TPE) adopts a more patient-centered approach, focusing on empowering patients and families to manage conditions, prevent complications, and improve quality of life. Led by trained healthcare providers, TPE emphasizes active patient involvement for a more lasting impact compared to traditional methods (6). However, despite positive effects on health behaviors, long-term benefits are often limited due to insufficient follow-up from patients’ perspectives (6).

In Africa, challenges such as poor adherence, high healthcare costs, and negative disease perceptions lead to inadequate disease management and treatment compliance, worsening outcomes for vulnerable families (1, 7). To address this, PAFLAR is implementing a TPE program with a “train the trainers” masterclass to ensure effective delivery and improve outcomes for children with JIA.

## Methods

### Implementing a TPE program

- Focus group:

A PAFLAR focus group was tasked with designing the TPE program and creating an implementation strategy.

A literature review was conducted to explore the research question: how to implement a therapeutic education program, leading to the identification of key steps. For the successful delivery of therapeutic patient education workshops, physicians and healthcare professionals must undergo specialized training (2). These education programs are designed to address both the needs prioritized by patients and those recognized by healthcare providers (4), using adaptable learning strategies based on patient feedback (4). Integrating educational technology plays a vital role by improving the accessibility of materials, personalizing content to align with the patient's learning preferences, fostering engagement, and accounting for the patient's limitations and strengths (2, 8). Moreover, involving family members in the care process is crucial for achieving comprehensive patient care (2). All these elements were considered in the development and implementation of the PAFLAR TPE program.

- Participant selection:

Participant selection was based on two main criteria: volunteering and demonstrating a commitment to actively participate in the program, as well as a strong dedication to implement the program in their respective environments with their patients.

- Program evaluation:

Evaluation was conducted at both the participant and patient levels by collecting feedback after the TPE workshops. Long-term evaluation focuses on the number of workshops conducted and the adherence of both patients and participants to the program.

## Results

### PAFLAR TPE project overview

The “Paediatric Society of the African League Against Rheumatism”: PAFLAR TPE project was launched in February 2023, outlining a targeted strategy to introduce TPE across African countries. The initiative began with an in-person TPE masterclass during the PAFLAR Conference in April 2023. Following this, specific virtual TPE workshops were organized for trainees under the guidance of experienced tutors, resulting in the development of five targeted workshops ready to be delivered to patients:

• The first workshop, titled “I Just Want to Understand My JIA,” aimed to provide patients and their parents with practical and straightforward information about the nature of the disease and its various manifestations, both articular and extra-articular.
• The second workshop, titled “I’m a Prince, I'm a Princess,” aimed to help children build and reinforce their self-esteem. The games were designed to show children that being sick is not a punishment and that being different is an asset. By the end of the session, children learned how to accept their disease, improve their self-esteem, and better integrate at school and with friends.
• The third workshop, titled “Physical Activity and JIA,” addressed the concerns that children with JIA and their parents often have about the potential harm sports might cause to affected joints. This uncertainty can be challenging for those who recognize the physical and mental health benefits of regular exercise but are unsure which activities are safe and suitable. Therefore, this workshop aimed to deepen participants’ understanding of the benefits and potential risks associated with physical activities and to help them choose appropriate sports that align with their health status and personal interests.
• The fourth workshop, titled “Treatment Adherence,” focused on the critical issue of treatment compliance. Addressing adherence issues early is essential to prevent complications and improve outcomes. Various factors influence children's adherence, including knowledge about medication, socioeconomic status, parental beliefs, attitudes toward side effects, and cultural influences. The games proposed in this workshop aimed to highlight the importance of adhering to treatment to avoid damage and disability in children and to help them find solutions for side effects and false beliefs. The workshop offered activities designed to enhance adherence and empower children to take charge of their treatment.
• The fifth workshop, titled “Your Superhero's Guide to Pain-Free Living,” focused on recognizing the various aspects of pain in JIA, including pain assessment and pain relief measures.

**Figure 1:**
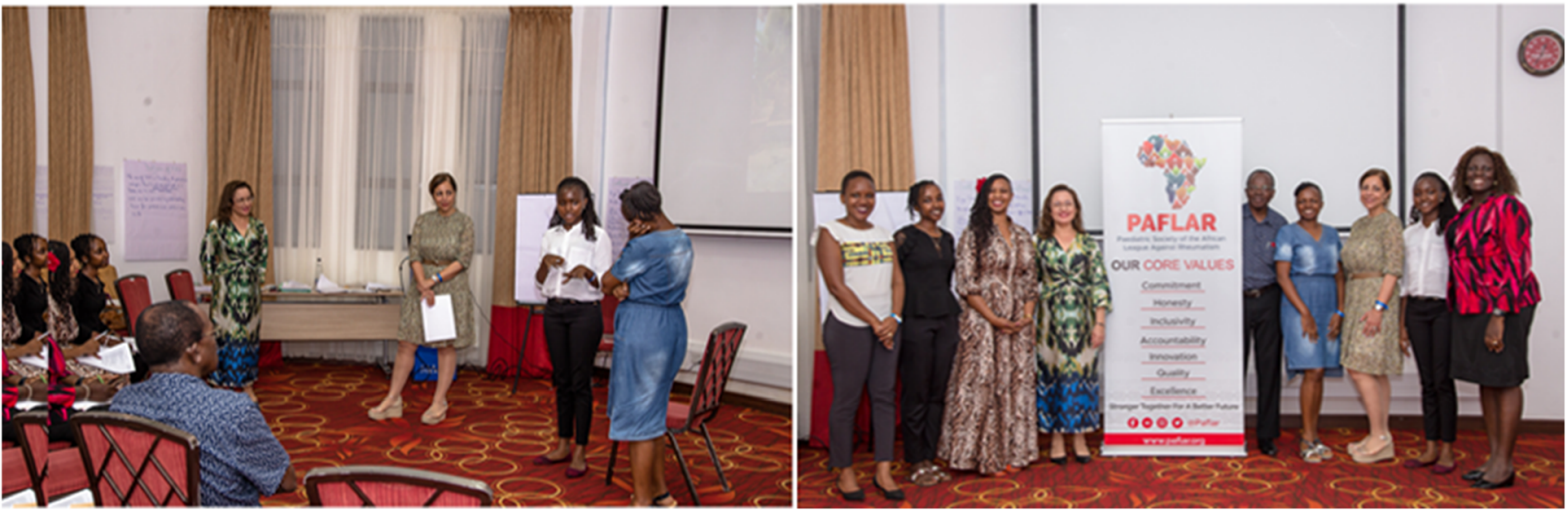
TPE masterclass conducted in mombasa Kenya during PAFLAR 2023 congress.

**Figure 2:**
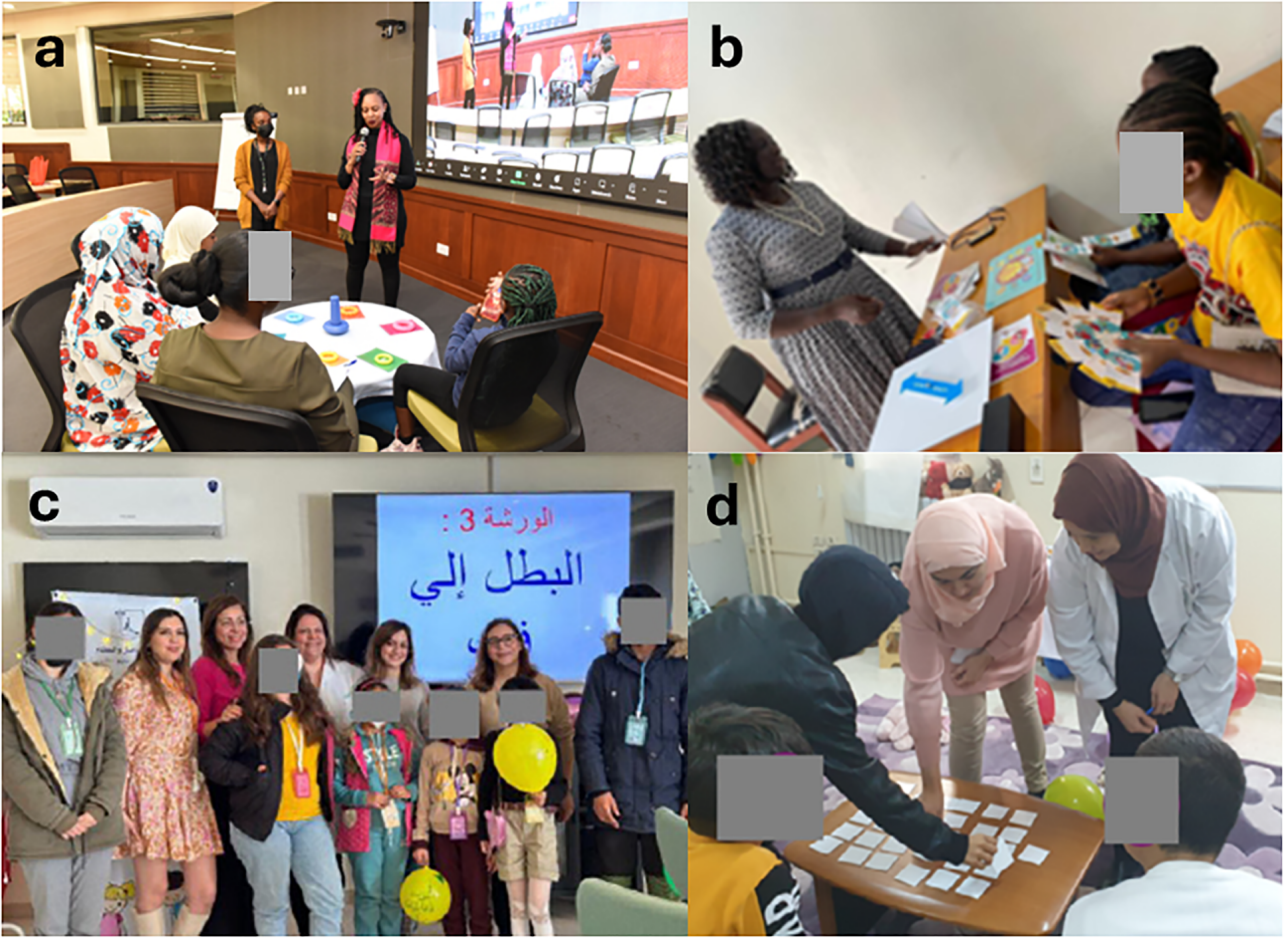
TPE workshops conducted in 4 pediatric rheumatology centers, respectively Kenya **(a)** Nigeria **(b)** and Tunisia **(c,d)**.

Kenya, Tunisia, and Nigeria actively participated in the masterclass (Figure 1). Professional designers crafted educational materials for the workshops, which were distributed to trainees in both English and Arabic (Supplementary Material). The materials were culturally and linguistically adapted for each country through close collaboration between designers and educators. Subsequently, healthcare professionals in these countries delivered the workshops to patients and collected feedback to relay to the tutors (Figure 2). After each workshop, children and their guardians completed a post-evaluation questionnaire to gauge satisfaction and gather insights for improving future sessions.

We analyzed the results of three TPE sessions held on different days for children diagnosed with JIA in two centers in Tunisia. Each child and their parents actively participated in these TPE sessions, which consisted of three 45 min workshops per session. The first workshop aimed to define JIA. The second workshop focused on identifying suitable physical activities for JIA patients. The final workshop centered on empowering children to manage pain through self-control techniques.

Before the TPE sessions, we assessed the participants’ baseline knowledge through anonymous pre-tests. The same questions were asked again at the end of each session as a post-test questionnaire. Additionally, parents were encouraged to complete a post-session assessment questionnaire. Responses to each question were recorded as follows: 0 (no correct response), 2 (a partially correct response), and 3 (a fully correct response). We then analyzed the scores of each question in the pre-TPE and post-TPE tests.

This initial endeavor was remarkably successful. Both parents and children have expressed a strong desire for additional workshops, emphasizing their need for a deeper understanding of the disease and improved management skills. This feedback underscores the significant value and impact of educational support for JIA within our community.

Patients who did not respond to both tests were excluded from the analysis. Nineteen children were enrolled in the study, with a mean age of 12.4 ± 3 years (ranging from 6 to 17 years). The male-to-female ratio was 1.11 (10 males, 9 females). The recorded JIA subtypes were: ERA in 47.4% (*n* = 9), oligoarticular in 15.8% (*n* = 3), polyarticular in 21.1% (*n* = 4), psoriatic arthritis in 5.3% (*n* = 1), and systemic onset in 10.2% (*n* = 2). Post-TPE test responses were received from 68.4% (*n* = 13) of parents.

Initially, the knowledge of JIA children about their condition was notably low, especially regarding the first workshop. The percentage of patients achieving a fully correct response in the pre-TPE sessions were: 21.1% (*n* = 4) for Workshop 1, 31.6% (*n* = 6) for Workshop 2, and 52.6% (*n* = 10) for Workshop 3. On average, children scored 3.84 out of 6 (64%) in the pre-session assessment questionnaire.

There was a significant improvement in the post-TPE evaluation. The percentage of patients achieving a fully correct response surged by 36.2% in the first workshop [reaching 57.9% (*n* = 11)], by 52.6% in the second [reaching 84.2% (*n* = 16)], and by 21.1% in the third [reaching 73.7% (*n* = 14)]. Among parents, none received a score of 0 in the post-session assessment test. Fully correct answers were noted by 36.8% (*n* = 7) for the question related to the first workshop, while 63.2% (*n* = 12) provided fully correct answers for the questions related to both the second and third workshops.

The qualitative analysis of the TPE project initiated by PAFLAR can be summarized as follows:

1. **Enhanced Healthcare Provider Skills**: Healthcare professionals received specialized training through in-person masterclasses and virtual sessions, gaining skills to effectively assess and meet patient needs.
2. **Improved Patient Empowerment**: Workshops empowered patients and their families with the knowledge needed to manage their conditions effectively. Education plans tailored to specific needs fostered a patient-centric approach, with different focuses in each country based on priority needs.
3. **Effective Virtual Support**: Post-training virtual support helped healthcare workers adapt and apply their skills locally, with trainers available for further consultation.
4. **Accessible Patient Education Materials**: A specialized firm designed educational tools that considered the learning styles, interests, and abilities of young patients, making materials more accessible, especially in Kenya and Nigeria where such initiatives were previously unavailable.
5. **Increased Collaboration and Family Involvement**: Workshops included family members in healthcare management, enhancing their understanding and strengthening collaborative care.
6. **Quality of Life Enhancement**: The project's goal is to enhance patients’ quality of life by equipping them with the necessary knowledge and skills to prevent complications and improve their overall well-being. Positive feedback was received following the initial workshops conducted at the four participating centers—two in Tunisia, one in Kenya, and one in Nigeria. Currently, a long-term study is underway to assess the impact of TPE on patient quality of life.
7. **Knowledge Dissemination**: Trained healthcare professionals served as ambassadors, spreading knowledge within their communities. Participants also received take-home brochures to reinforce the lessons learned and enhance ongoing patient care.

## Discussion

Numerous studies have demonstrated the impact of patient education on health-related outcomes, yet the lasting effects often remain unexplored due to insufficient ongoing evaluation from the patients’ perspectives (9–11). In Africa, despite the committed efforts of healthcare professionals to treat thousands of patients with chronic inflammatory rheumatism, the outcomes have frequently fallen short (7). This shortfall is attributed to several factors, including low adherence to treatment protocols, the prohibitive costs of healthcare in the absence of insurance, and prevailing negative attitudes towards these conditions (4, 6). As a result, management and medication compliance are typically suboptimal, placing undue burdens on already disadvantaged families and communities, leading to preventable morbidities and fatalities.

Patient education stands as a fundamental component of healthcare, significantly enhancing health status and quality of life (10). Therapeutic education empowers patients by equipping them with essential skills to take an active role in managing their health (3, 5, 12). Through tailored therapeutic education programs that are sensitive to their socio-economic circumstances, patients gain a deeper understanding of their conditions (6). This understanding fosters acceptance and more effective disease management, aligning with the goals of TPE (6, 12).

Implementing TPE workshops in African children with JIA faces several barriers and challenges, crucially stemming from diverse cultural, economic, and infrastructural differences across the continent (7, 12). Accessibility remains a primary challenge, with many areas lacking the basic healthcare infrastructure needed to deliver consistent and effective TPE. Economic constraints further limit access to essential educational materials and trained healthcare professionals. Additionally, cultural perceptions of illness and treatment can significantly affect the acceptance and effectiveness of TPE, necessitating culturally adapted educational strategies to improve engagement and outcomes (3–5, 12). Collaboration with local organizations and advocacy groups in pediatric rheumatology can play a crucial role in overcoming these barriers.

The impact of different educational materials such as videos, brochures, comics, and TPE workshops can vary greatly depending on the setting and culture (3, 5, 12). Visual materials like videos and comics may be more effective in conveying complex information in an engaging and understandable manner, particularly for children (3, 5). They can break language barriers and simplify understanding, crucial in regions with high illiteracy rates. However, the success of any educational tool also depends on its cultural relevance and the extent to which it resonates with local beliefs and practices. TPE workshops that involve family members and are adapted to local contexts tend to be more effective, as they not only educate but also build community support systems around patients, enhancing adherence to treatment and ongoing management strategies (6).

The challenges PAFLAR faces in effectively and consistently implementing the TPE program include maintaining sustained motivation among educators, increasing patient awareness of the importance of TPE workshops to improve their participation rate, and mobilizing resources to train additional educators in more African countries.

PAFLAR aims to actively expand its TPE programs, with an initial focus on increasing awareness among healthcare providers about the critical role of TPE in improving patient quality of life, particularly targeting JIA and aiming for a broader reach across countries lacking specialized care. This effort underscores PAFLAR's commitment to advance patient education in underserved areas. Moreover, the plan to extend TPE applications beyond JIA and to adapt and deploy these educational models to other diseases, tailoring programs to address the unique needs and challenges of different conditions. This strategic initiative aims to develop flexible educational resources that can significantly impact disease management and patient outcomes across various healthcare settings.

## Conclusion

In conclusion, PAFLAR has taken the initial step in implementing a TPE program for JIA across three countries, representing different regions of Africa. The TPE program offers a promising alternative to traditional patient education methods, significantly improving patient care, empowering healthcare providers, and advancing healthcare systems. Successfully implementing this ambitious project in challenging environments requires overcoming existing barriers with determination, ambition, and optimism.

## Data Availability

The original contributions presented in the study are included in the article/Supplementary Material, further inquiries can be directed to the corresponding author.
