# Supplementary material for: “Breaking barriers: empowering African youth through implementation of a therapeutic patient education program for juvenile idiopathic arthritis”
Source: Front Pediatr. 2024 Dec 3;12:1479857. doi: 10.3389/fped.2024.1479857 (PMC11653180; doi:10.3389/fped.2024.1479857)
Supplement: Supplementary file 1 [file Presentation1.pdf]

# “Breaking barriers: empowering African youth through implementation of a therapeutic patient education program for juvenile idiopathic arthritis”

Supplementry material

# State of the Art

# Children from 4 to 6 years old

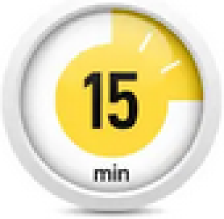


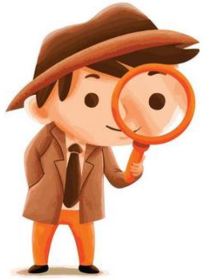

## "The pain detective's adventure: Discover how pain is expressed during JIA"

### Materials Needed

A Selection of photos illustrating various expressions of pain by children

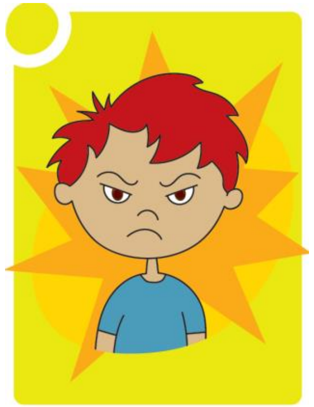

Contracted face

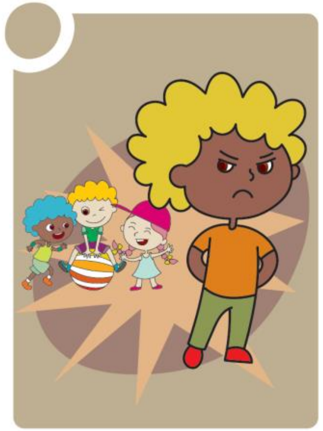

Refusal to play

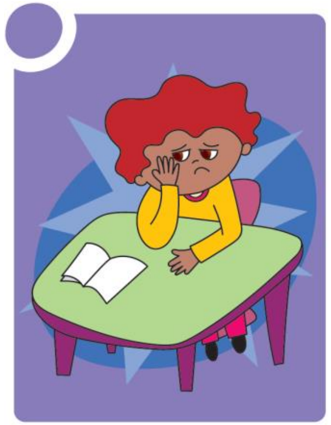

Disinterest in school

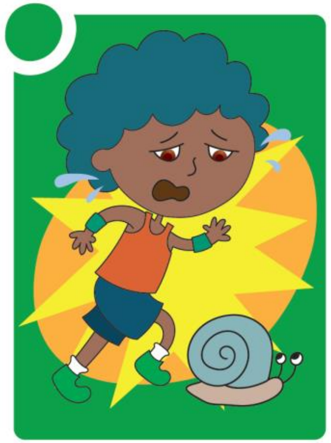

Slowness of movement

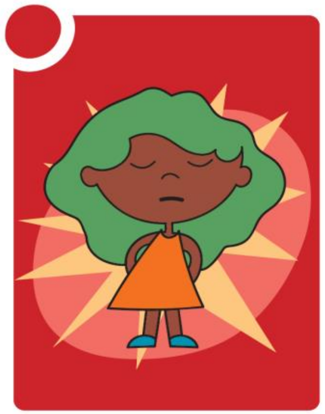

Inexpressive child

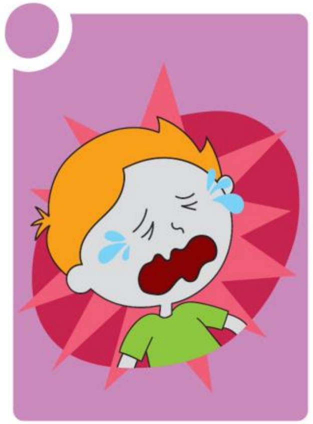

Crying

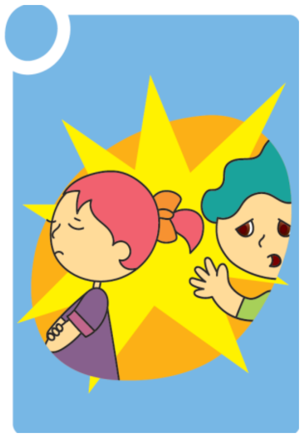

Refusal to speak

**State of the Art** Children over 6 years old

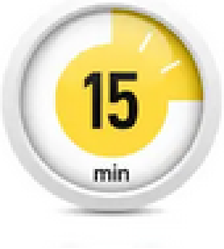


# "Pain Scale Adventure: Discovering Your Pain Superpower"

## Materials Needed:

Variety of pictures, each depicting different situations

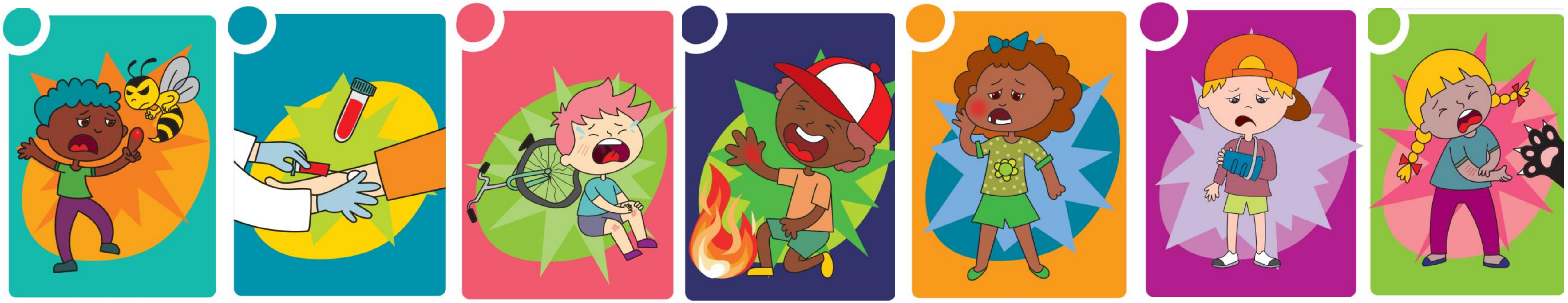

# Learning activity & Solutions

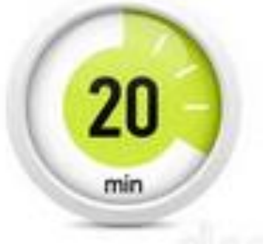


## "Pain Management Explorers"

**Materials needed :** A spinning wheel with different situations that may or may not correspond to a solution for managing pain

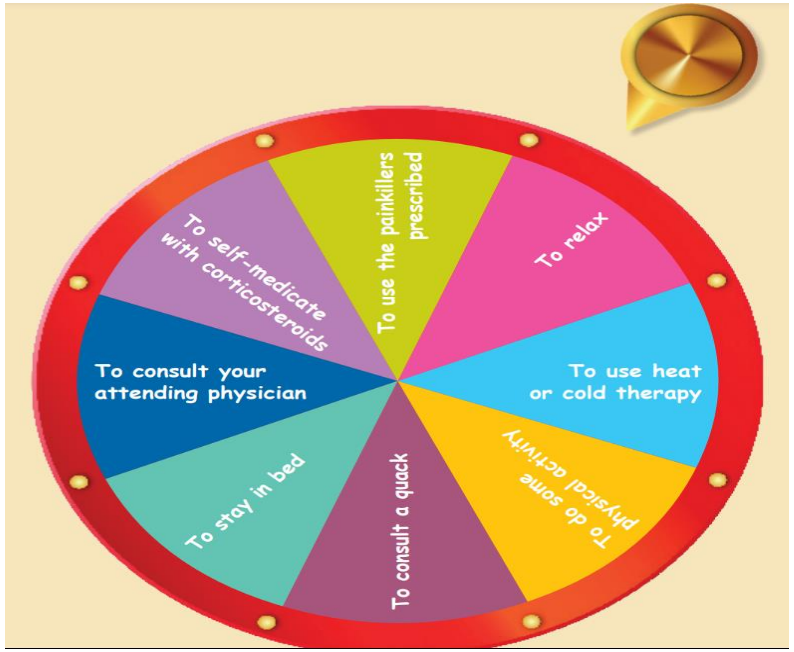

# Handout

## A guide including pain management strategies for pain associated with JIA

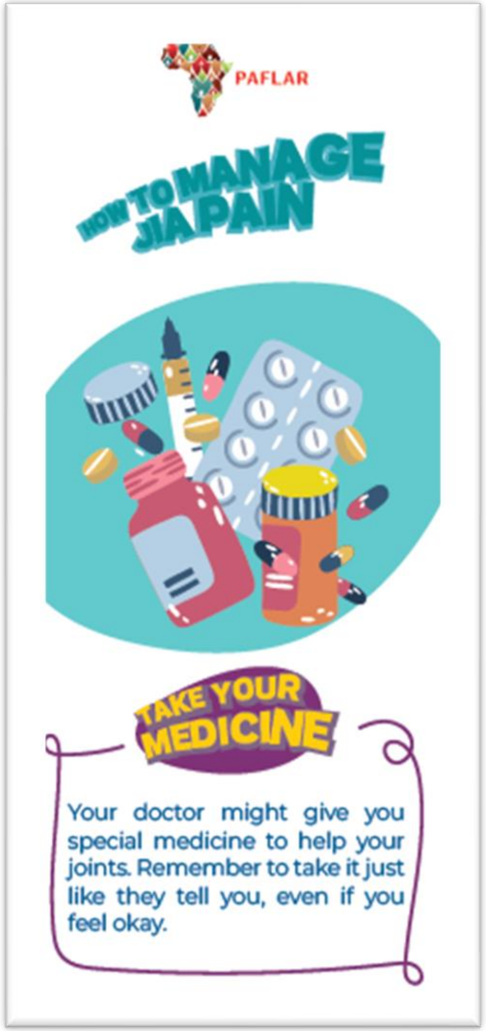


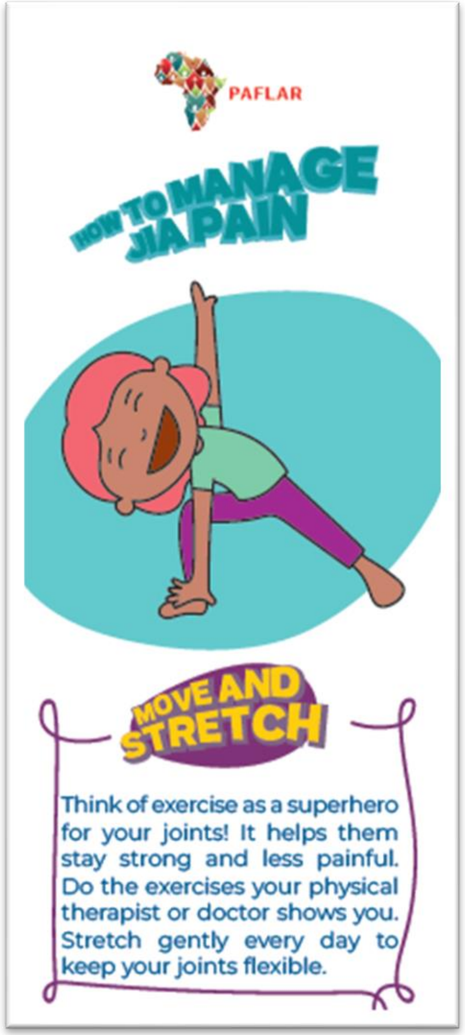


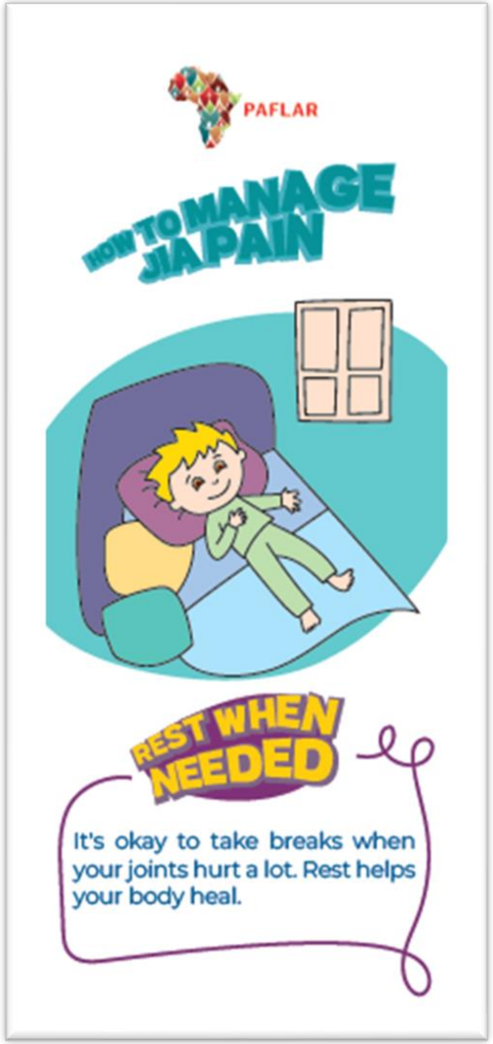


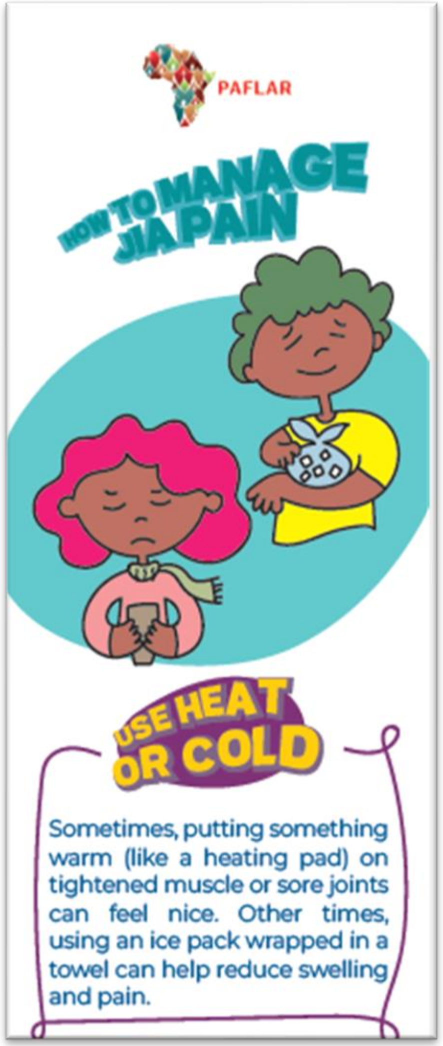


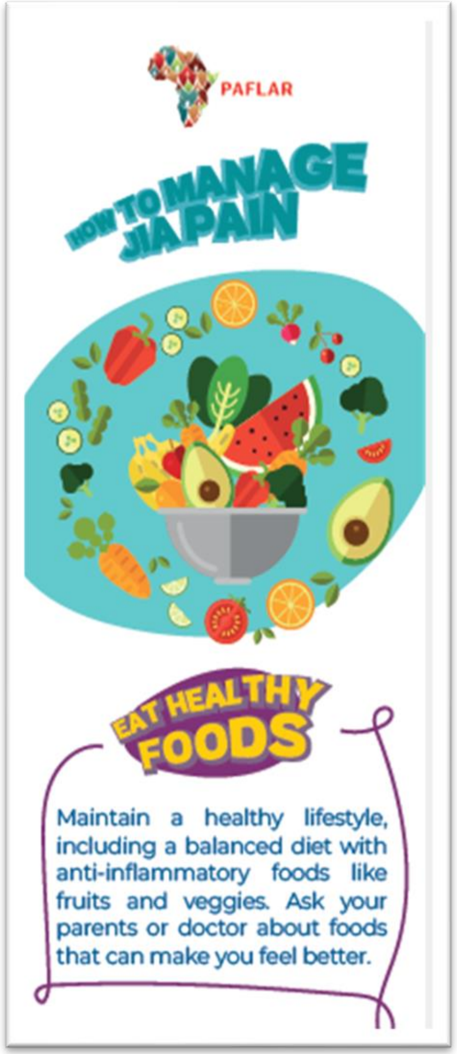

# Handout

## A guide including pain management strategies for pain associated with JIA

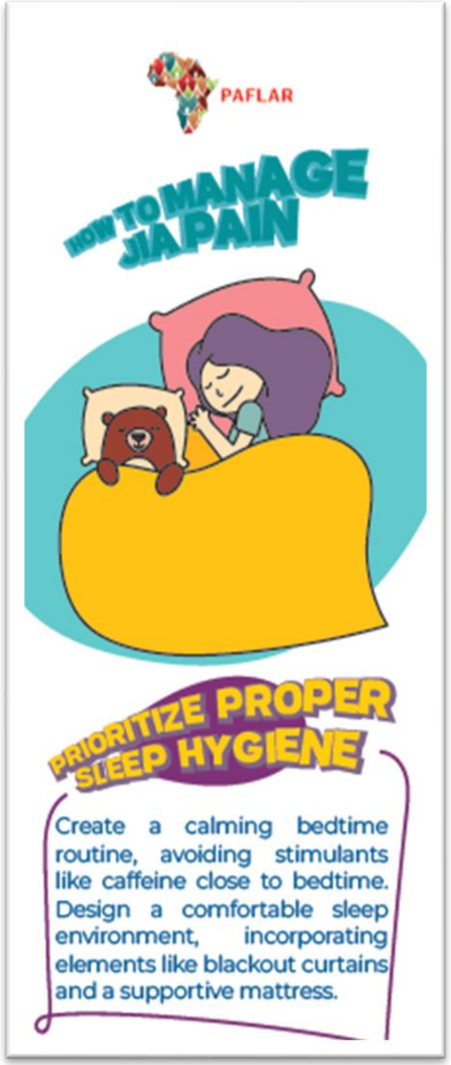


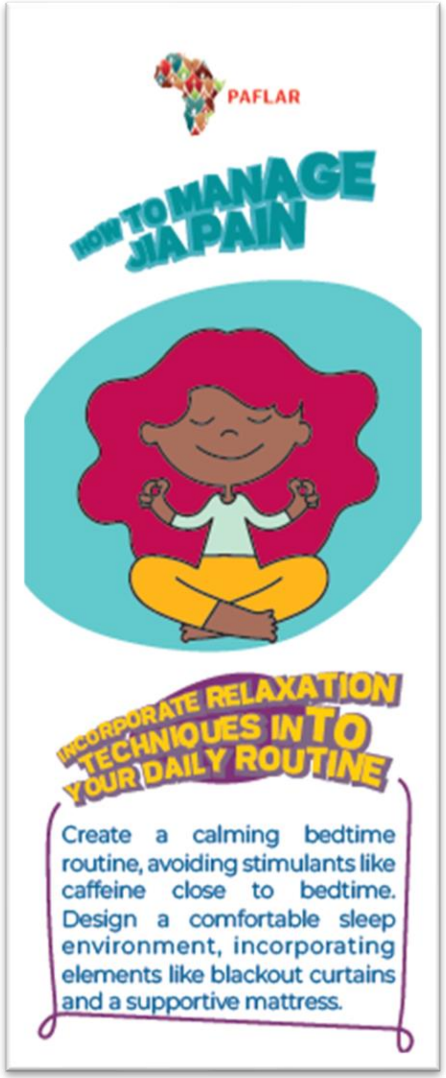


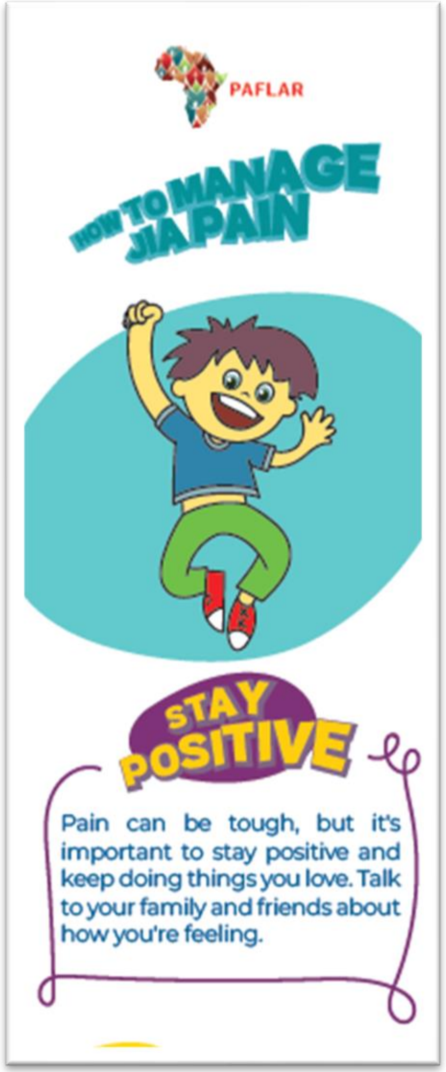


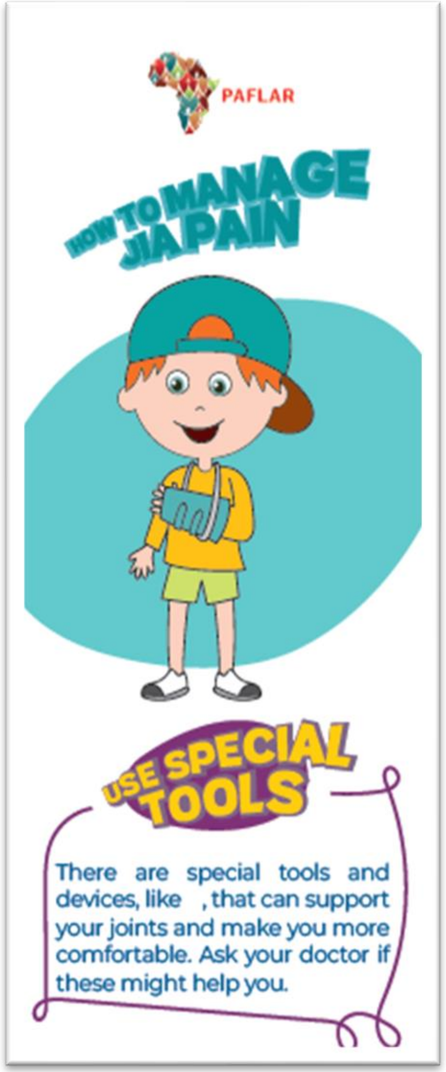


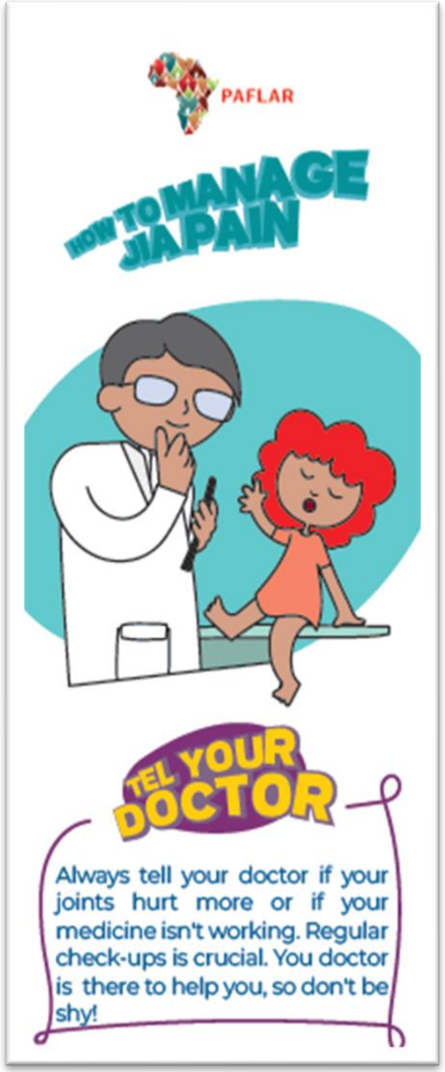

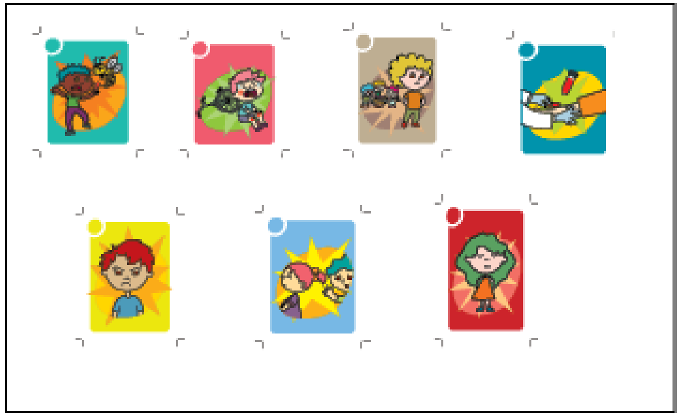

Workshop 2: "Understanding Pain"

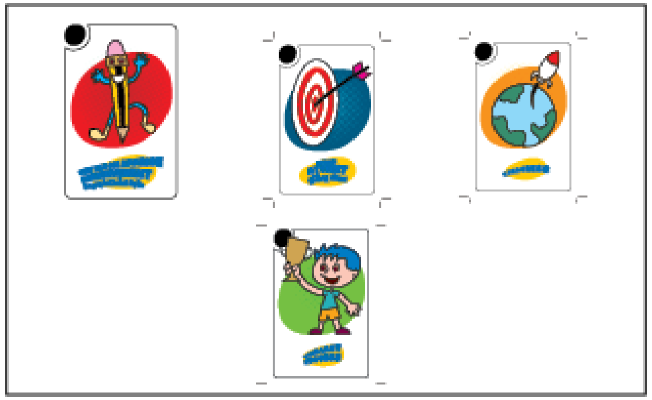

Workshop 4: "How to Live Well with my Disease"

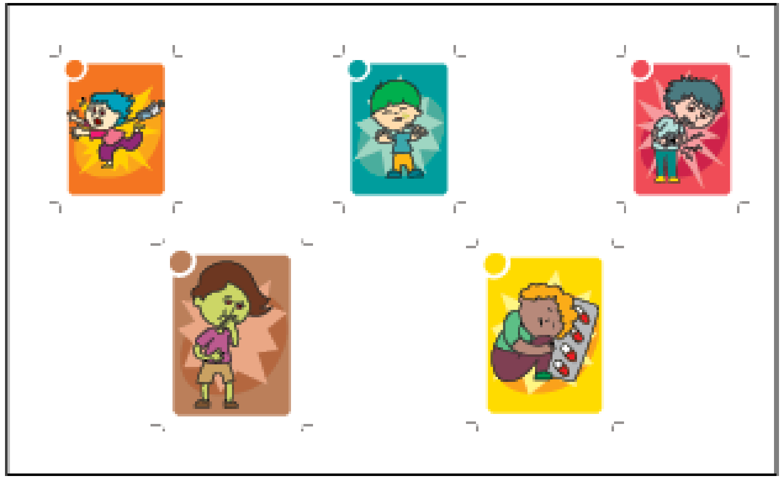

Workshop 5: "Treatment Adherence"

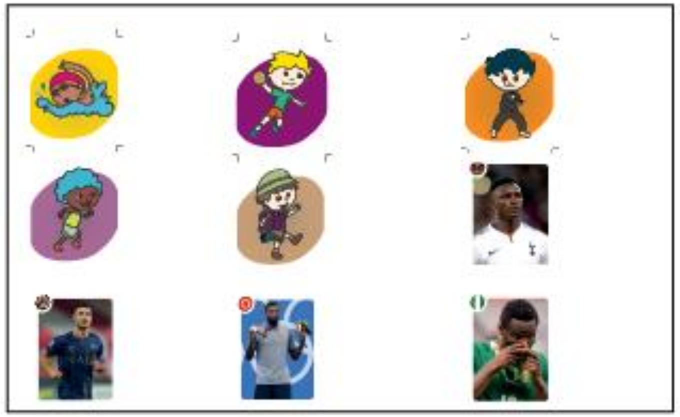

Workshop 3: "Physical Activity and JIA"

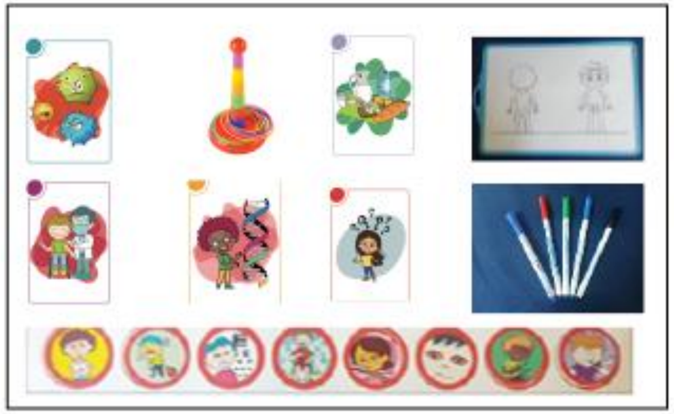

Workshop 1: "What is Juvenile Idiopathic Arthritis?"
